# Microbial Biotransformation to Obtain New Antifungals

**DOI:** 10.3389/fmicb.2015.01433

**Published:** 2015-12-24

**Authors:** Luiz F. Bianchini, Maria F. C. Arruda, Sergio R. Vieira, Patrícia M. S. Campelo, Ana M. T. Grégio, Edvaldo A. R. Rosa

**Affiliations:** ^1^Xenobiotics Research Unit, School of Health and Biosciences, The Pontifical Catholic University of ParanaCuritiba, Brazil; ^2^Faculty of Dentistry, School of Health and Biosciences, The Pontifical Catholic University of ParanaCuritiba, Brazil

**Keywords:** microbial biotransformation, biocatalysis, bioconversion, metabolism, antifungals

## Abstract

Antifungal drugs belong to few chemical groups and such low diversity limits the therapeutic choices. The urgent need of innovative options has pushed researchers to search new bioactive molecules. Literature regarding the last 15 years reveals that different research groups have used different approaches to achieve such goal. However, the discovery of molecules with different mechanisms of action still demands considerable time and efforts. This review was conceived to present how Pharmaceutical Biotechnology might contribute to the discovery of molecules with antifungal properties by microbial biotransformation procedures. Authors present some aspects of (1) microbial biotransformation of herbal medicines and food; (2) possibility of major and minor molecular amendments in existing molecules by biocatalysis; (3) methodological improvements in processes involving whole cells and immobilized enzymes; (4) potential of endophytic fungi to produce antimicrobials by bioconversions; and (5) *in silico* research driving to the improvement of molecules. All these issues belong to a new conception of transformation procedures, so-called “green chemistry,” which aims the highest possible efficiency with reduced production of waste and the smallest environmental impact.

## Introduction

The search for new molecules has forced the pharmaceutical industry to modernize its synthetic processes. Such modernization has occurred with the adoption of new techniques, such as miniaturization, nanotechnology, microdosing, chemometrics, and high-throughput analysis (Koh et al., 2003). Another aspect of modernization, so-called “Green Chemistry,” requires new synthetic processes with the highest possible efficiency, resulting in reduced production of waste and the smallest environmental impact (Tucker, 2006).

More than 20,000 molecules with antibiotic activity that are produced by microorganisms have been described since the discovery of penicillin by Sir Alexander Fleming; however, only a small fraction of them are clinically useful due to their toxicity. Since the 1980s, a decline in the discovery of new molecules has been observed (Murphy, 2012).

The antifungal agents available on the market act on different targets such as ergosterol synthesis, chitin synthesis, glucan synthesis, nucleic acid synthesis, protein synthesis, microtubule synthesis, or as inhibitors of squalene epoxidase or ergosterol disruptors (Kathiravan et al., 2012). However, although there is substantial variability in the mechanisms of action and despite the technical advances, the development of new antifungal drugs persists considering the co-evolution of resistance mechanisms. Between 2006 and 2010, only one antifungal was approved for use, a natural echinocandin that was chemically changed by a semi-synthetic route (Chen et al., 2011). In the last 30 years, among the 28 new naturally occurring molecules, only three semi-synthetic molecules that underwent chemical changes have been authorized for clinical use (Newman and Cragg, 2012).

It is estimated that 25% of the world's population presents some episode of superficial mycosis and the mortality rate associated with invasive fungal infections frequently exceeds 50%, even with the available antifungal medications. This amount corresponds to approximately 1.5 million deaths annually (Brown et al., 2012). Even under prophylactic use with antifungals, some changes in epidemiological features have been reported in more aggressive mucormicoses in patients using voriconazole, as well as the rampant development of resistance to azole by *Aspergillus* spp. (Perfect et al., 2014).

Echinocandins and allylamines are more modern drug classes approved, but they date back to the 1970s and 1980s, respectively (Odds, 2003). Echinocandins have been used as the treatment of choice for systemic candidoses and are effective for strains resistant to azoles (Zimbeck et al., 2010; Eschenauer et al., 2014); however, a mutation in the *FKS* gene increases the resistance of *Candida* spp. to echinocandins (Zimbeck et al., 2010; Beyda et al., 2012). A multicenter study demonstrated that *C. glabrata* and *C. krusei* have lost their susceptibility to caspofungin (24 and 52%, respectively) and that other common *Candida* species are rapidly losing their susceptibility to echinocandins (Zimbeck et al., 2010).

Even assuming great advances in synthetic chemistry, biotransformation (or biocatalysis) remains the most cost-effective path to discover new pharmaceuticals (Zaks and Dodds, 1997). Special attention should be given to the significant number of drugs produced by microorganisms or by interactions with the host from which they were isolated. Both cases contribute to the idea that biotransformation processes shall expand significantly in the future (Newman and Cragg, 2012). Nevertheless, there are some questions regarding biotransformation that have to be addressed: (1) How can structural changes occur in a way to make the processes more time- and cost-efficient? (2) How can the biological activity of products be enhanced by optimizing the pharmacokinetic/pharmacodynamic (PK/PD) properties and safety? (3) How may a supplier guarantee large-scale drug production under good quality practices? (Bauer and Brönstrup, 2014).

With the appropriation of concepts from White Biotechnology and Green Chemistry, this review aims to assess the technological advances in the development of microbial biotransformation products with antifungal activity.

## Microbial metabolism vs. microbial biotransformation

Natural products compose more than 2/3 of antibiotics used in the medical/dental/veterinary practice (Schmitz et al., 2013). Thus, it is not an erroneous statement to assume that active substances from plants or those isolated from microorganisms are the simplest way to search for new molecules. If they have antifungal potential, some criteria must be observed (Barrett, 2002): (1) Do they present novel mechanisms of action or any useful known mechanisms? (2) Is it possible to obtain clinical proof with good biological activity? (3) Is it possible to change the molecule to make it a tolerable drug? A good example of a bioactive molecule obtained from plant extracts is eugenol, which is extracted from *Eugenia caryophillis* (Indian clove). It is a phenylpropanoid that presents considerable fungicidal activity *in vitro* against *C. albicans*, and, unlike fluconazole, is also effective against *C. krusei* and *C. glabrata* (Ahmad et al., 2010).

In addition to plants, microorganisms have provided some bioactive molecules with remarkable antimicrobial activity, especially in the last two decades.

For fungal infections, the glycopeptide occidiofungin A is produced by *Burkholderia contaminans* MS14 and presents great antifungal activity against pathogens of plants and animals (Lu et al., 2009). Its mechanism of action has not been elucidated, but it is assumed that it differs from the known classes and can bypass fungal resistance problems (Tan et al., 2012). Benanomicin A and benanomicin B are fermentatively produced by the cultivation of *Actinomadura spadix* MH193-16F4 and are broad-spectrum antimicrobials against various fungi including endemic and opportunistic pathogens (Kumagai et al., 2008).

Although there remains a myriad of naturally occurring secondary metabolites to be evaluated and discovered, microbial biotransformation has emerged as an important tool for obtaining novel structural analogs or to improve the pharmacokinetic parameters of other substances (Parshikov et al., 2000; Borges et al., 2008; Baydoun et al., 2014).

It is important to emphasize that metabolism and biotransformation are distinct systems of molecular processing. Microbial metabolism is composed of two major processes; primary metabolism, which is responsible for cellular function, and secondary metabolism, which uses pre-existing metabolic pathways to produce substances from endogenous intermediates to allow better adaptation of the organism to the environment (Keller et al., 2005; Brakhage, 2013). Primary metabolism consists of reactions associated with energy generating, biomass production, and essential cell components. Events such as glycolysis, oxidative phosphorylation, and the Calvin-Benson cycle (in algae and photosynthesizing bacteria) are examples of typical sets of reactions of primary metabolism.

In contrast, secondary metabolism involves important events for adaptation to environmental conditions. In general, it generates low molar mass metabolites that are not essential for growth but that offer some advantages and may sometimes have medical/veterinary/agricultural/industrial importance. They include antibiotics, pigments, anti-tumor agents, etc. They have unusual structures and are normally synthesized during the late phase of cell growth (Ruiz et al., 2010).

Regarding to antibiotics, it is estimated that actinomycetes are responsible for 70–80% of all molecules produced by secondary metabolism. Different species belonging to the genus *Streptomyces* produces important antibiotics as chloramphenicol, streptomycin, macrolides, and rifampicin, among others (Raja and Prabakarana, 2011). A recent review regarding to this theme presented new perspectives about the optimization in the production of actinomycetes-derived antibiotics (Antoraz et al., 2015).

Biotransformation, sometimes inaccurately called “xenobiotic metabolism,” is responsible for minor structural modifications in exogenous substances by enzyme systems that lead to the formation of molecules with relatively greater polarity (Asha and Vidyavathi, 2009; Pervaiz et al., 2013). Phenomena such as stereoselective hydroxylation, epoxidation, and oxidation are common reactions attributed to biotransformation processes and have been reported to occur in fungi (Farooq et al., 2002; Choudhary et al., 2005, 2007, 2009, 2010; Al-Aboudi et al., 2009). It is a mechanism that microorganisms developed to adapt to environmental changes and it is useful in a wide range of biotechnological processes (Crešnar and Petric, 2011).

**Figure 1:**
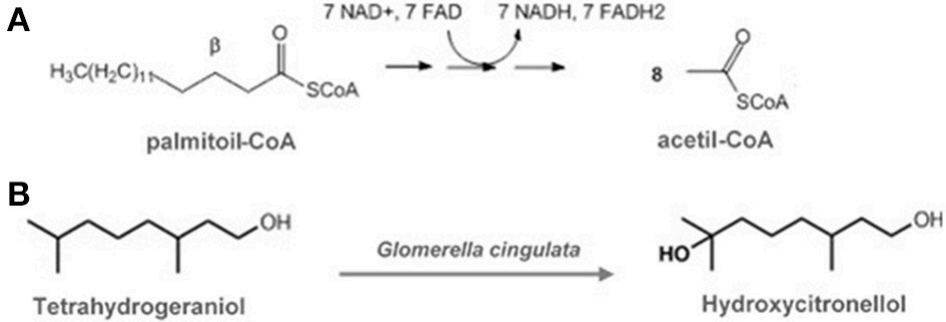
**(A)** Typical multi-step metabolic reactions, with deep alterations on carbon skeleton (in this case, fatty acid oxidation). **(B)** Typical one-step biotransformation reaction, with minor punctual alterations on carbon skeleton (Adapted from: Hegazy et al., 2015).

One of the most remarkable features of biotransformation reactions is the maintenance of the original carbon skeleton after obtaining the products. During metabolism, the carbon atoms are transferred to other molecules with different chemical functions (Figure 1).

Biotransformation reactions may involve various events such as the formation of stable intermediates, which may be devoid of toxic or pharmacologic activity. Sometimes, short-lived reactants may also be generated. Further, biotransformation reactions can result in chemically stable compounds with desired pharmacological activity (Fura, 2006).

The use of microbial biotransformation is part of a new movement named White Biotechnology, which is an emerging field of modern biotechnology that serves industry. It employs living cells (animals, plants, algae, filamentous fungi, yeast, actinomycetes, and bacteria), as well as enzymes produced by these cells during the generation of products of interest.

Wisely, Venisetty and Ciddi (2003) presented nine practical advantages in the use of microbial systems as models for drug metabolism:

1. The low-cost and facility to maintain stock cultures of microorganisms;
2. Procedures with large number of strains are simple repetitive processes;
3. The concentrations of parental molecules used (generally ranging from 0.2 to 0.5 g/L) are much higher than those employed in other cell or tissue models;
4. Novel products can be isolated with new or different activities;
5. There is a possibility to predict the most favored metabolic reactions;
6. The models can be scaled up easily for the preparation of metabolites for pharmacological and toxicological evaluation;
7. These models can be utilized in synthetic reactions where tedious steps are involved;
8. In most cases, relatively mild incubation conditions are used;
9. The models can be useful in cases where regio- and stereo-specificity are involved, becoming molecular handling more easily achieved by biotransformation than by synthetic chemistry.

With regards to these last two statements, obtaining an antimicrobial may become critically laborious if only chemical procedures are employed, even for semi-synthetic compounds (Wild, 1994; Claes et al., 2013). In this context, microbial biotransformation becomes an attractive resource.

Living cells can be used in their original state (wild strains) or improved to work as “cell factories” to produce enzymes or consumer goods (Carballeira et al., 2009). Despite their potential, the number and diversity of applications is still modest when considering the wide availability of microorganisms, the large number of reactions that they can achieve and the fact that biotransformation reactions are considered economically and ecologically competitive (Borges et al., 2009).

## Biotransformation by whole cells vs. immobilized enzymes

It is insufficient to know the microbial biotransformation pathways to establish whether they are economically useful; rather, it is necessary to define reproducibility at a production scale. This assumption demands some concern regarding raw materials (the molecule to be biotransformed and the microorganism/enzyme responsible for the reaction), equipment (bioreactors), and the necessary technology to be employed in the purification of the products (downstream processing). In addition, it is imperative to take into account the lowest possible production of pollutants and the highest possible enantiomeric purity of the final product.

The criteria above are focuses of Green Chemistry that have allowed the construction of chiral chemical building blocks that lead to the development of enantiopure products obtained under mild reaction conditions at physiological pH and temperature, using water as reaction milieu and environmentally friendly catalysts (enzymes or cells). The obtained products are usually multifunctional molecules that exhibit highly chemo-regio-stereoselective activity (Borges et al., 2009; Muñoz Solano et al., 2012).

Immobilized enzymes are used for the conversion of molecules in many industrial fields, although they are preferably directed to simple catalytic processes. For the production of antimicrobials, the applicability of individualized enzymes in *stricto sensu* biotransformation processes is still incipient and little explored (Banerjee, 1993a,b; Mujawar, 1999; Takimoto et al., 2004; Hormigo et al., 2010). Possibly, this may result from the functional complexity required, which is readily attainable by living organisms.

An important enzyme feature that has not been properly explored is their promiscuity, which is the ability of enzymes to catalyze different reactions with distinct catalytic mechanisms to create new pathways (Wu et al., 2010). If we obtained a new molecule with remarkable antifungal properties but with inappropriate characteristics for ADMET (administration, distribution, metabolism, excretion, and toxicity), it is possible to “improve” its structure by enzyme promiscuity. For this, *in silico* technologies favor technical development processes, predicting PK/PD characteristics and increasing the use of immobilized enzymes in the synthesis of pharmaceuticals.

Some complex processes of anti-proliferation production via enzymatic biotransformation are already known. A good example is the biosynthesis of griseofulvin from malonyl-CoA using purified enzymes (Cacho et al., 2013). Several phases including aldol condensation, cyclization, halogenation, and oxidation are enzyme-mediated steps that can be reproduced without the need of a living organism.

Penicillin G acylase (PGA) is one of the most relevant and widely used biocatalysts for the industrial production of β-lactam semisynthetic antibiotics (Srirangan et al., 2013). Such an enzyme may be bulk produced as heterologous PGA in competent strains such as *Escherichia coli* ATCC® 11105™ (Erarslan et al., 1990) or *Bacillus badius* PGS10 (Rajendran et al., 2011).

Considering the processes using whole cells, it is assumed that fungi are the organisms most commonly used to obtain natural metabolic products and for biotransformation reactions (Borges et al., 2009), even for the procurement of antifungal drugs; however, microorganisms from other kingdoms may also conduct dedicated biotransformation processes in order to obtain antifungal molecules (Table 1).

**Table 1:** **Antifungal molecules obtained by microbial biotransformation**.

**Antifungal chemical**	**Biotransformer microorganism**	**Parental chemical**	**Antifungal activity**	**Citation**
bEPA		Eicosapentaenoic acid	*Botrytis cinerea*	Bajpai et al., 2008
			*Colletotrichum capsici*	Bajpai and Kang, 2007
bDHA	*Pseudomonas aeruginosa* NRRL-B-18602	Docosahexaenoic acid	*Fusarium oxysporum*	Bajpai et al., 2009b
bEFA		Hydroxifatty acids: ricinoleic acid, linoleic acid, eicosadienoic acid, etc	*Fusarium solani Phytophthora capsici Rhizoctonia solani Sclerotinia sclerotiorum*	Bajpai et al., 2006
2,3-dihydrotrichostatin A	*Streptomyces venezuelae* YJ028	Trichostatin A	*Saccharomyces cerevisiae*	Park et al., 2011
Ethyl p-hydroxycinnamate	*Aspergillus niger*	Ethyl p-methoxycinnamate	*Candida albicans*	Omar et al., 2014
9-keto-(-)-vasicine	*Aspergillus braziliensis* ATCC®16404™	(-)-vasicine	*Candida albicans*	Gopkumar and Mugeraya, 2010
	*Penicillium notatum* ATCC®36740™ *Rhizopus arrizus* ATCC®10260™			
	*Trametes versicolor* ATCC®20869™			
5-p-menthene-1,2-diol	*Alternaria alternata* NRRL20593	α-phellandrene	*Candida* spp.	İşcan et al., 2012
	*Aspergillus alliaceus* NRRL317			
	*Aspergillus flavus*			
	*Botrytis cinerea* AHU9424			
	*Devosia riboflavina* NRRL-B-784			
	*Fusarium culmorum*			
	*Fusarium heterosporium* DSM62719			
	*Fusarium solani* ATCC®1284™			
	*Kluyveromyces lactis* NRRL-Y-8279			
	*Neurospora crassa* N23 and N24			
	*Phanerochaete chrysosporium* ATCC®24725™			
	*Saccharomyces cerevisae* ATCC®9763™			
	*Yarrowia lipolitica* NRRL-Y-B423			
Oxylipins	*Pseudomonas aeruginosa* 42A2	Hydroxifatty acids: ricinoleic acid, linoleic acid, oleic acid, palmitic acid, etc.	*Verticillium dhaliae*	Martin-Arjol et al., 2010
			*Macrophonia phaesolina*	
			*Arthroderma uncinatum*	
			*Trycophyton mentagrophytes*	
Biotransformed galbonilides I and II	*Streptomyces halstedii* ATCC®55964™	Galbonolides A and B	*Candida albicans*	Shafiee et al., 1999
			*Cryptococcus neoformans*	

The use of whole cells is advantageous once they present all the needed enzymes and cofactors in adequate concentrations and energy status. These favorable conditions may modulate the activity of multienzymatic complexes and contribute to increase conversion rates (Restaino et al., 2014).

The obtaining of biotransformation products may be conducted using co-cultivation of two or more distinct entities. It has been proposed by Wu et al. (2015), who reported the increased obtaining of bioactive molecules during the co-cultivation of *Streptomyces coelicolor* A3(2)M145 (actinomycete) and *Aspergillus niger* N402 (fungus). According to the authors, “growth in microbial communities or interactions between different microorganisms is the next logical step in the search for new molecules.”

Another interesting approach involves the possibility of heterologous expression of biotransformation-related cytochrome P450 enzymes from actinomycetes in bacteria to obtain new antimycotic derivates (Kumagai et al., 2008). It becomes useful for biotransformation procedures in large scale producing plants where large bioreactors are employed.

It has been reported that pyrethrosin, a germacrane sesquiterpene lactone commonly found in *Chrysanthemum cinerariaefolium* Visiani (Asteraceae), can be converted to several new molecules by *Cunninghamella elegans* NRRL1392 and *Rhizopus nigricans* NRRL1477 (Galal, 2001). Such filamentous fungi are able to completely deplete pyrethrosin, transforming it into five more polar metabolites that are very active against *Cryptococcus neoformans* (IC_50_ = 25–35.0 μg.mL^−1^) and *Candida albicans* (IC_50_ = 10–30 μg.mL^−1^).

The search for new antifungal molecules via microbial biotransformation involves not only purified precursor molecules, but may encompass complex substrates that can be bioconverted into extracts rich in active substances.

Biotransformation products of cabbage-crude extracts (*Brassica oleracea* var. *capitata*) processed by *Pseudomonas syringe* pv. T1 showed promising inhibitory activity for several *Candida* spp., with values close to those obtained for amphotericin B (Bajpai et al., 2011). *Pectobacterium atrosepticum* Pepto-A, a Gram-negative plant pathogen, can also be produced from cabbage extracts to yield anti-*Candida* compounds (Bajpai et al., 2012a).

Another *Pectobacterium* species, *P. carotovorum* subsp. *carotovorum* 21, also presented the ability to biotransform compounds found in cabbage (Bajpai et al., 2010a) and in tomatoes (*Solanun esculentum*) to generate others with remarkable activity against *Candida* spp. (Bajpai et al., 2012b,c).

Biotransformation has been successfully utilized as a tool to generate pharmaceutical compounds from natural products. Through this process, ethyl p-methoxycinnamate (EPMC) was extracted from *Kaempferia galanga* (a Malaysian plant) and was transformed using *Aspergilus niger* to ethyl p-hydroxycinnamate (EPHC) (Omar et al., 2014). Looking at antimicrobial activities, EPHC has a potential inhibitory effect against *C. albicans* that is better than the effect by EPMC. This highlights the possibility of increasing antifungal value to other known antifungal molecules.

Studies in this area shall be directed toward the diversification of substrates combined with the numerous species of biotransforming microorganisms. This can thus result in the expansion of the antifungal library. In a recent review, a number of molecules with antimicrobial activity derived from monoterpenes have been described after biotransformation processes by various microorganisms (Bhatti et al., 2014).

## Endophytic fungi produce antimicrobials by biotransformation

Despite the substantial worldwide diversity, the discovery of new families of bioactive molecules is surprisingly declining, driving the need to bioprospect new sources (Joseph and Priya, 2011). Following such a thought, a group with promising potential for new discoveries in the biotransformation area is that of endophytic fungi. In recent years, endophytic fungi have garnered great interest.

They are organisms that can grow either intra or extracellularly in the tissues of higher plants in a clear mutualistic relationship without causing any symptoms. Evidence shows that they are a rich source of bioactive natural products. Active metabolites of endophytics show positive actions as antibiotics, immunosuppressives, anti-helminthics, antioxidants, and anticancer drugs (Pimentel et al., 2011).

There is a noticeable increase in the rate of resistance to antimicrobials, which is, at least in part, related to the insufficient number of effective molecules and the small amount of new antimicrobial agents in development, probably due to unfavorable investment returns. In this context, the endophytic fungi present themselves as an attractive alternative to modify the current paradigm (Molina et al., 2012).

However, although a promising field, some problems cannot be ignored in studies with endophytes. Special attention is recommended in face of:

1. The high nonspecific toxicity of some antimicrobials already obtained;
2. The fact that fungi tend to not produce toxic substances against themselves, resulting in antimicrobial compounds with moderate antifungal activity without potential as a medicament or pesticide;
3. The difficulty in large scale production of certain antimicrobial compounds in artificial culture media;
4. The biosynthesis and regulation of production of antimicrobials from endophytes are partially or totally unknown (Yu et al., 2010).

It is important to mention that many of the products related to endophytics are derived from their secondary metabolism, and do not necessarily have a biotransformation background. In some cases, it is not clear whether the final product is the result of secondary metabolism or biotransformation. In spite of this last finding, it is common sense amongst investigators devoted to biotransformation that these fungi present vast potential (Pimentel et al., 2011).

Table 2 presents some molecules with antifungal activity that are produced exclusively by endophytic fungi via secondary metabolism. As such, organisms that usually live in harsh conditions in the presence of numerous natural compounds are expected to be exceptional biotransforming entities (Shibuya et al., 2003, 2005; Agusta et al., 2005; Fu et al., 2011; Maehara et al., 2011; Huang et al., 2015; Khoyratty et al., 2015). Although endophytic fungi are promising organisms for biotransformation processes, their ability and potential to produce antimicrobial molecules remain unexplored.

**Table 2:** **Antifungal molecules obtained from secondary metabolism of endophytic fungi**.

**Antifungal chemical**	**Endophytic fungi**	**Origin**	**Antifungal activity**	**Citation**
Camptothecin	*Colletotrichum* sp.	*Artemisia annua*	Various human and plant pathogens	
Periconicin A and B				
Phomol				Guo et al., 2008
Pyrrocidines A and B	*Acremonium zeae* NRRL13540	*Zea mays*	*Aspergillus flavus*	
			*Fusarium verticillioides*	
Sordaricin	*Xylaria* sp. PSU-D14	*Garcinia dulcis*	*Candida albicans*	Pongcharoen et al., 2008
Lactone multiplolides A and B	*Xylaria multiplex* BCC 1111	Unidentified Thai tree	*Candida albicans*	Boonphong et al., 2001
7-amino-4-methylcoumarin	*Xylaria* sp. YX-28	*Gynkgo biloba*	*Aspergillus niger*	Liu et al., 2008
			*Candida albicans*	
			*Penicillium. expansum*	
Griseofulvin	*Xylaria* sp. F0010	*Abies holophylla*	*Blumeria graminis* f. sp. *hordei*	Park et al., 2005
			*Corticium sasaki*	
			*Magnaporthe grisea*	
			*Puccinia recondite*	
Chaetomugilin A and D	*Chaetomium globosum*	*Ginkgo biloba*	*Mucor miehei*	Qin et al., 2009
Cytosporone B and C	*Phomopsis* sp. ZSU-H76	*Excoecaria agallocha*	*Candida albicans Fusarium oxysporum*	Huang et al., 2008
(-)-Mycorrhizin A (+)-Cryptosporiopsin	*Pezicula* spp.	Various German trees	*Euratium repens*	Schulz et al., 1995
			*Mycatypha micraspara*	
			*Ustilaga vialacea*	
Pestalachlorides A, B, and C	*Pestalotiopsis adusta* (L416)	Unidentified Chinese tree	*Fusarium culmorum*	Li et al., 2008
			*Gibberella zeae*	
			*Verticillium albo-atrum*	
Emodin Hypericin	*Thielavia subthermophila*	*Hypericum perforatum*	*Aspergillus niger*	Kusari et al., 2008, 2009; Kusari and Spiteller, 2011
			*Candida albicans*	
Brefeldin A	*Cladosporium* sp.	*Quercus variabilis*	*Aspergillus niger*	Wang et al., 2007
			*Candida albicans*	
			*Epidermophyton floccosum*	
			*Microsporum canis*	
			*Trichophyton rubrum*	
Cytochalasin D 2-hexyl-3-methyl-butanodioic acid	*Xylaria* sp	*Palicourea* marcgravii	*Cladosporium cladosporioides*	Cafêu et al., 2005
Ethyl 2,4-dihydroxy-5,6-dimethylbenzoate Phomopsilactone	*Phomopsis cassia*	*Cassia spectabilis*	*Cladosporium sphaerospermum*	Silva et al., 2005
Asperfumoid	*Aspergillus fumigatus* CY018	*Cynodon dactylon*	*Candida albicans*	Liu et al., 2004
Fumigaclavine C				
Fumitremorgin C				
Helvolic acid				
Physcion				
2,6-diOH-2-methyl-7-(prop-1E-enyl)-1-benzofuran-3(2H)-one Ergosterol peroxide	*Verticillium* sp.	*Rehmannia glutinosa*	*Fusarium* sp.	You et al., 2009
			*Rhizoctonia* sp.	
			*Septoria* sp.	

## Candidate molecules for biotransformation

One point of concern when evaluating the feasibility of producing antifungals via microbial biotransformation is the choice of parental molecules to be modified. Such chemicals must not be toxic or inhibitory to the biotransforming organism. However, this can be circumvented if the concentrations during fermentation processes remain inferior to those considered as inhibitory.

Accumulated data has revealed that some classes of molecules seem to be more ready to undergo biotransformation and generate antifungals.

Some unsaturated fatty acids have shown interesting results. Bajpai et al. (2009a, 2010b) have conducted extensive reviews regarding this matter and reported that *Pseudomonas aeruginosa* NRRL-B-18602 PR3 produced mono-, di-, and tri-hydroxy fatty acid derivatives from unsaturated fatty acids with recognized antifungal properties. This bacterium can convert oleic acid to 7,10-dihydroxy-8(E)-octadecenoic acid, an anticandidal compound (Hou and Forman, 2000).

Other unsaturated fatty acids can also undergo oxidation in the presence of bacteria (*Pseudomonas* sp. 42A2 or *Bacillus megaterium* ALA2 NRRL-B-21660) or plants (*Colocassia antiquorum*) to produce mono-, di-, and tri-hydroxy fatty acids with antifungal potential such as 15,18-dihydroxy-14,17-epoxy-5(Z),8(Z), 11(Z)-eicosatrienoic acid, 17,20-dihydroxy-16,19-epoxy-4(Z),7(Z),10(Z),13(Z)- docosatetraenoic, 9,12,13-trihydroxy-(E)-octadecenoic acid, 12,13,16-trihydroxy- 9(Z)-octadecenoic acid, and 12,13,17-trihydroxy-9(Z)-octadecenoic acid (Masui et al., 1989; De Andrés et al., 1994; Hou, 1996; Hou et al., 1997; Hosokawa et al., 2003a,b).

Based on the mechanism proposed for (Z)-9-heptadecenoic acid (Avis and Bélanger, 2001), the most probable antifungal mechanism of action must involve the disruption or disintegration of the plasma membrane caused by a hydrostatic turgor pressure within the cell resulting in the release of intracellular electrolytes and proteins (Carballeira, 2008).

Another class of molecules with a promising outlook are sterols. This class of substances has been evaluated in relation to their ability to be biotransformed for many years (Mahato and Mukherjee, 1984; Mahato and Garai, 1997; Holland, 1999; Malaviya and Gomes, 2008; Bhatti and Khera, 2012; Donova and Egorova, 2012). Surprisingly, there are few investigations on the production of antifungals, despite the fact that many compounds such as 24-amino-lanosterol, 24-amino-cholesterol, and 24-amino-cholesterol-N-sulfate possess potent antifungal activities against *Candida* spp., *C. neoformans*, and *Trichophyton mentagrophytes* (Chung et al., 1998).

Preliminary studies have shown that steroids and steroidal lactones biotransformed by *Cunninghamella* spp. produce metabolites with leishmanicidal activity (Choudhary et al., 2006; Baydoun et al., 2014). It is reasonable that such metabolites act on the synthetic pathway of ergosterol in *Leishmania* spp. We speculate that these putative mechanisms of action may be extrapolated to fungi, which should encourage investigators to drive their efforts toward this problem.

Alkaloids may also be converted into antifungal compounds. As part of an extensive program which aimed the discovery and development of antimicrobials from higher plants, Orabi and colleagues conducted a series of experiments in order to obtain antifungals from sampangine, an alkaloid found in the West African tree *Cleistophathis patens* (Annonaceae) (Orabi et al., 1999). Their results showed that *Beauveria bassiana* ATCC®7159™, *Doratomyces microsporus* ATCC®16225™, and *Filobasidiella neoformans* ATCC®10226™ produced the 4′-O-methyl-β-glucopyranose conjugate, while *Absidia glauca* ATCC®22752™, *Cunninghamella elegans* ATCC®9245™, *Cunninghamella* sp. NRRL5695, and *Rhizopus arrhizus* ATCC®11145™ produced the β-glucopyranose conjugate. Both metabolites presented significant *in vitro* activity against *C. neoformans,* but were inactive against *C. albicans* (Orabi et al., 1999).

The same group published interesting results about the biotransformation of the synthetic antifungal alkaloid benzosampangine (Orabi et al., 2000). They showed that *Absidia glauca* ATCC®22752™, *Cunninghamella blakesleeana* ATCC®8688a™, *Cunninghamella* sp. NRRL5695, *Fusarium solani* f. sp. *cucurbitae* CSIH#C-5, and *Rhizopogon* species ATCC®36060™ each produced a β-glucopyranose conjugate of benzosampangine. Such a substance possesses good *in vitro* antifungal activity against *C. albicans, Aspergillus fumigatus* (MIC = 0.39 μg.mL^−1^; amphotericin B: 0.78 μg.mL^−1^ and 0.39 μg.mL^−1^, respectively), and *C. neoformans* (MIC = 1.56 μg.mL^−1^; amphotercin B, 0.39 μg.mL^−1^). The authors emphasized that microbial biotransformation is reliable and produces significant quantities of metabolites. In addition, they showed that alkaloids could be converted into conjugate metabolites with increased antifungal activity.

## *In silico* predictive improvement of bioprocessable molecules

*In silico* is the term used to define experimentation carried out in computers. In turn, *in silico* pharmacology is a large growing area that helps to develop molecular arrangements using dedicated software to capture, analyze and integrate biological and medical data (Ekins et al., 2007). The use of *in silico* techniques allows the prediction of the pharmacokinetic aspects of absorption, distribution, biotransformation and excretion of new substances. It can be assumed that *in silico* projections of new molecules allow the prioritization of chemicals to be tested, identifying hazard and risk assessment (Kulkarni et al., 2005). The computer-assisted simulations based on pharmacological and biological data reduce time and costs during the screening of new substances once they cease to categorize undesirable molecules with improper characteristics during the early stages of discovery.

In addition, it is possible to evaluate the coupling of molecules to their possible targets. Using databases of 3D molecular structures, it is possible to anticipate connections between new molecules and possible binding sites. Numerous substances have been evaluated by this method, and it was possible to propose important features of some of them as the mechanism of action of antifungal piranocoumarins and antibacterial xanthone derivatives (Do et al., 2015).

CaCYP51 is a sterol 14-α demethylase that binds the CYP51 substrates lanosterol and eburicol in *C. albicans. In silico* techniques based on the molecular structure of CaCYP51 have allowed the development of new azoles by replacing the side chains provided in the simulation. The new azoles showed excellent antifungal activity *in vitro* with broad spectrum (Che et al., 2009). The development of new molecules *in silico* largely depends on the molecular recognition of possible couplings between the drug and microorganisms. Therefore, once elucidated, the mechanism of action of an antifungal obtained by biotransformation will allow the simulation of molecules with improved pharmacokinetic characteristics and may accelerate the development of potential drugs (Rask-Andersen et al., 2011).

## Perspectives

The prospection of new molecules, principally from endophytic fungi, is a vast field of study with huge potential for growth. However, consistent efforts are necessary to achieve results. Professionals from the fields of biotechnology, pharmacology, computer sciences, engineering and some other related areas have to work closely together to explore possible binding sites for new and existing molecules. The use of *in silico* analyses anticipate investigations in a timesaving manner and reduce the demands for inputs and even animals used in the initial tests. These features drive future research toward the development of new and feasible technologies within the Green Chemistry guidelines connected to the principle of the 3Rs (replacement, reducing, refinement) for the use of experimental animals (Russell and Burch, 1959).

A reasonable research design for the prospecting of new antifungal drugs under the above stated conditions involves the following:

1. The *in silico* analysis of existing molecules for possible new couplings to different targets;
2. The *in silico* identification of necessary changes in the precursor molecule for effective coupling on targets;
3. In the absence of new discoveries, the biotransformation of various candidate substrates for obtaining new molecules;
4. The recognition of which organisms or isolated enzymes may modify structural precursor molecules to yield enantiomerically pure antifungal molecules based on the theory of enzyme promiscuity;
5. The *in silico* evaluation of toxicity for newly developed antifungal molecules;
6. The process of scaling up with environmentally friendly inputs to test the economic viability.

Once the goals outlined above are achieved, the investigator is ready to conduct clinical trials to confirm the PK/PD properties, security, and all other steps necessary for commercialization of the drug.

It is important to notice that with the advent of *in silico* technologies, laboratory tests tend to reduce their margin of error, and therefore, save both environmental and financial resources. However, they primarily accelerate new discoveries to minimize the impact of developed resistance to existing drugs.

Therefore, it is necessary to sequence and deposit the genomes of microorganisms with biotransformation capacity in databases. In addition, further study on the molecular structure of possible targets in pathogenic fungi is mandatory. This will provide subsidies to find coupling regions for the developed molecules.

Currently, we have high cost and low return as factors related to the decline in the development of new antimicrobial drugs. There is no other way to achieve such a market if the costs of research are not reduced and production is enabled in a sustainable way.

## Funding

This review was supported entirely by the Pontifical Catholic University of Parana, to whom the authors are grateful.

### Conflict of interest statement

The authors declare that the research was conducted in the absence of any commercial or financial relationships that could be construed as a potential conflict of interest.
